# Lack of Informed and Affirming Healthcare for Sexual Minority Men: A Call for Patient-Centered Care

**DOI:** 10.1007/s11606-024-08635-8

**Published:** 2024-02-02

**Authors:** Kevin Hascher, Jessica Jaiswal, Caleb LoSchiavo, Jerel Ezell, Danika Duffalo, Richard E. Greene, Amanda Cox, Wanda M. Burton, Marybec Griffin, Tejossy John, Benjamin Grin, Perry N. Halkitis

**Affiliations:** 1 Feinberg School of Medicine, Northwestern University, Chicago, IL 60611 USA; 2 Department of Family and Community Medicine, University of Alabama, Birmingham School of Medicine, Birmingham, AL 35294 USA; 3 Center for Interdisciplinary Research on AIDS, Yale University School of Public Health, New Haven, CT 06510 USA; 4 Center for Health, Identity, Behavior and Prevention Studies (CHIBPS), Rutgers School of Public Health, Newark, NJ 07102 USA; 5 Heersink School of Medicine, University of Alabama at Birmingham, Birmingham, AL 35294 USA; 6 Department of Health Behavior, Society and Policy, Rutgers School of Public Health, Piscataway, NJ 08854 USA; 7 Department of Community Health Sciences, UC Berkeley, Berkeley, CA 94720 USA; 8 Center for Cultural Humility, UC Berkeley, Berkeley, CA 94720 USA; 9 School of Medicine, Creighton University, Phoenix, AZ USA; 10 Department of Medicine, New York University Grossman School of Medicine, New York, NY 10001 USA; 11 Culverhouse College of Business, University of Alabama, Tuscaloosa, AL USA; 12 Capstone College of Nursing, University of Alabama, Tuscaloosa, AL 35401 USA; 13 Department of Primary Care, Kansas City University College of Osteopathic Medicine, Kansas City, MO 64106 USA; 14 Department of Biostatistics and Epidemiology, Rutgers School of Public Health, Piscataway, NJ 08854 USA; 15 Department of Urban-Global Public Health, Rutgers School of Public Health, Newark, NJ 07102 USA

## Abstract

**Background:**

Sexual minority men (SMM) face severe health inequities alongside negative experiences that drive avoidance of medical care. Understanding how SMM experience healthcare is paramount to improving this population’s health. Patient-centered care, which emphasizes mutual respect and collaboration between patients and providers, may alleviate the disparaging effects of the homophobia that SMM face in healthcare settings.

**Objective:**

To explore how SMM perceive their experiences with healthcare providers and how care can most effectively meet their needs.

**Design:**

Semi-structured qualitative interviews focused on healthcare experiences, pre-exposure prophylaxis (PrEP), and HIV-related beliefs were conducted between July and November 2018.

**Participants:**

The study included a sample of 43 young adult SMM (ages 25–27), representing diverse socioeconomic, racial, and ethnic backgrounds, in New York City.

**Approach:**

Researchers utilized a multiphase, systematic coding method to identify salient themes in the interview transcripts.

**Key Results:**

Analyses revealed three main themes: (1) SMM perceived that their clinicians often lack adequate skills and knowledge required to provide care that considers participants’ identities and behaviors; (2) SMM desired patient-centered care as a way to regain agency and actively participate in making decisions about their health; and (3) SMM felt that patient-centered care was more common with providers who were LGBTQ-affirming, including many who felt that this was especially true for LGBTQ-identified providers.

**Conclusions:**

SMM expressed a clear and strong desire for patient-centered approaches to care, often informed by experiences with healthcare providers who were unable to adequately meet their needs. However, widespread adoption of patient-centered care will require improving education and training for clinicians, with a focus on LGBTQ-specific clinical care and cultural humility. Through centering patients’ preferences and experiences in the construction of care, patient-centered care can reduce health inequities among SMM and empower healthcare utilization in a population burdened by historic and ongoing stigmatization.

## INTRODUCTION

A syndemic of biopsychosocial and systemic factors explains the myriad health inequities that sexual minority (e.g., gay, bi, or queer-identifying) men face in comparison to heterosexual peers,^1–7^ with inequities amplified for sexual minority men (SMM) of color due to multiple and intersecting minority stressors across individual, interpersonal, institutional, and systemic levels.^8–14^ Emerging adulthood is an especially critical period for the health of SMM who are affected by a variety of health disparities while navigating newfound idependence.^15^ Experiences in healthcare settings, including interactions with clinicians, play an important role in shaping the health and health behaviors of SMM.^15^ For example, negative healthcare experiences are consistently associated with healthcare avoidance and sexual identity nondisclosure, inhibiting SMM from receiving appropriate care,^16–25^ as well as with unmet health needs, worse health outcomes, and lower satisfaction with care.^23–28^ The pervasiveness of negative healthcare experiences may be attributed in part to limitations in medical and health-related education;^29–34^ with LGBTQ-specific training often as brief as 1–2 h, clinicians are left unprepared to care for sexual and gender minority patients.^30,98^

Patient-centered care (PCC) offers a way to improve healthcare experiences and health outcomes by centering patients’ unique experiences, needs, and desires.^35–37^ PCC utilizes focused approaches to understand and address the specific outcomes that matter to each individual. Through practices such as shared decision-making,^38,39^ PCC promotes greater satisfaction with care, fewer unmet health needs, improved health outcomes, and reduced medical mistrust,^40–44^ without increasing costs.^45^ Thus, incorporating PCC in clinical practice can help clinicians better deliver affirming and comprehensive healthcare.^46^

The extant literature regularly underscores LGBTQ people’s need for PCC approaches,^47–56^ with positive correlates including increased identity disclosure, enhanced patient satisfaction, and improved HIV treatment adherence.^56–58^ However, few studies explore experiences with and perspectives about PCC among LGBTQ patients, with diverse samples of SMM notably underrepresented.^56,59–62^ With the growing recognition of the patient experience as a salient measure of healthcare quality, there is a pressing need for studies that address it.^99^ Thus, the current study utilizes a qualitative approach to illuminate the healthcare–related experiences and perceptions of SMM to better understand how affirming, sensitive, and high-quality care can be delivered to SMM.

## METHODS

### Study Design and Sample

Data for this analysis are drawn from the Health-Related Beliefs Sub-Study, a mixed-methods study nested within a longitudinal study following a socio-demographically diverse cohort of young SMM and transgender women in New York City. Methods for the Health-Related Beliefs Sub-Study and parent P18 Studies have been described extensively elsewhere; briefly, P18 participants (at baseline: assigned male at birth, 22–23-year-old, HIV-negative, NYC-area residents who reported recent male sex partners) completed biannual study visits including computer-based and interviewer-administered assessments and HIV/STI testing.^2,15,70,71^ HIV-negative parent study participants were invited to enroll in the Health-Related Beliefs Sub-Study, with 43 participants (Table 1) in the qualitative study component. Throughout the duration of the parent study, a small percentage of participants transitioned and identified as transgender women or non-binary. Transwomen and non-binary participants were retained in the study and thus included in the sub-study presented here. All participants provided written informed consent and all activities were approved by the New York University IRB.

**Table 1:** Sample Characteristics of Participants (*n* = 43)

	% (*n*)
*Age*	
Reported as* M (SD)*	25.86 (0.74)
*Race/ethnicity*	
Black	25.6 (11)
White	25.6 (11)
Hispanic/Latino	27.9 (12)
Asian	20.9 (9)
*Education*	
High school/GED or less	20.9 (9)
Some college, no degree	18.6 (8)
Bachelor’s or graduate degree	60.5 (26)
*Total annual income, past year*	
<$15,000	18.6 (8)
$15,000–24,999	20.9 (9)
$25,000–44,999	32.6 (14)
≥$45,000	23.3 (10)
Missing	4.7 (2)

### Data Collection

The second author (PI of the study) and a research assistant, both trained in qualitative methods, designed a semi-structured interview guide that explored beliefs about and experiences with health and healthcare, including questions on HIV, PrEP, and interactions with clinicians and healthcare systems. The interview guide contained questions regarding participants’ disclosure of their sexuality to healthcare providers, sexual behavior, and experiences of stigma with clinicians. Participants were not asked about their respective clinician’s demographic details. Between July and November 2018, trained research staff completed interviews that typically lasted about 1 h (range, 30–75 min). While saturation was reached at 35 interviews,^63^ 43 were completed for representativeness.

### Data Analysis

Recordings were professionally transcribed and quality-checked by research assistants. To protect confidentiality, participants were assigned pseudonyms. The four team members implemented a multiphase, systematic coding method, consisting of initial coding, organizing codes and subcodes, and then extracting and reexamining codes to identify their relationships. Three of the team members (including the first author) coded and extracted data from each interview; the first and second authors closely reviewed the analysis to resolve any differences in interpretation. Additional methods of establishing trustworthiness included credibility and confirmability techniques such as periodic external audits and peer debriefing.^64,65^ Data was organized using ATLAS.ti 8.

## RESULTS

Three main themes were identified in the results: (1) SMM perceive their providers to have low LGBTQ+ competence; (2) SMM express a strong desire for PCC; and (3) SMM realize that LGBTQ+ affirming providers facilitate PCC. Broadly, participants explained that their providers were often uncomfortable with health issues that affect SMM and they often lacked the language to discuss such topics, resulting in microaggressions. As a result, participants expressed a strong desire for PCC, particularly from LGBTQ+-identifying clinicians.

### Theme 1: SMM Perceive Their Providers to Have Low LGBTQ Health Competence

Participants described clinicians who seemed ill-equipped to provide care that aptly accounted for their social and cultural identities, primarily through insufficient medical knowledge. Many participants described how their primary care providers (PCPs) were lacking interpersonal and communication skills.^100,101^ For example, Eric described his PCP’s inability to provide HPV-related information or referrals:

*[My provider] doesn’t seem to know anything about gay stuff, and I actually during a P18 study like two years ago, did one of the butt swabs and found out I have four strains of HPV… so I went to go see him subsequent, I don’t know, a couple months after that. It’s like, ‘Oh, by the way. I did this voluntary study, NYU, yadda, yadda,. Swabbed my butt. HPV.’ … And he’s Googling and he’s like scra[mbling] – and he’s like, ‘I think I need to refer you to someone.’ He was very confused. And, and he’s like, ‘Well, maybe I should check it.’ And I was like, ‘Okay. All right. Maybe you should.’ You know, he didn’t really know what to say or do.* [Eric, White, 26]

Benjamin echoed these sentiments when describing his experience seeking emergency care, expressing his discontent with heteronormativity in healthcare:

*Unfortunately, not everybody’s trained to look into a butthole or to judge what a normal rash is from maybe something else. [I got a rash] and I was panicking. So, I did go to the hospital with the intent of, ‘Hey, can you take a look at my crack? It might need some help. I’m not sure what’s going on.’ At first, I did get refused. It was a woman doctor. She was like, ‘Oh, no. I think you have to go to a special facility.’ I’m like, ‘Well, this is the emergency room and this might be an emergency. I don't know. I need you to tell me if it’s an emergency, you know, to seek further treatment.’ It’s always been kind of a gray area when you bring up sex in the hospital and you’re not straight... as a gay male, it’s almost still taboo – even in 2018 – at least in the medical field, in the hetero medical field.* [Benjamin, Latinx, 25]

Another major aspect of theme 1 was participants’ descriptions of providers’ inability to communicate appropriately about sexuality. Participants stated that providers’ language regarding their sexual identity or behavior was often stigmatizing. For example, Javier described his discomfort with his provider’s framing of sexual positioning:

*It was very casual and it wasn’t I would say in an offensive way, but it was just more so like ‘Are you taking it or receiving it?’ something like that I believe. I was just like, ‘That’s –‘ I don't know. It just felt weird for him to say it like that. I don't know if you would use the word like top or bottom. I can’t think of a better way personally but, for me, it just felt weird…I’m not used to talking to someone like a doctor about that, but it was just an uncomfortable, awkward moment.* [Javier, Latinx, 26]

Heteronormativity was similarly apparent in communication beyond sexual health, as told by Daniel:

*I have a husband, so I’ve had doctors, when they ask me about my life, they say wife, ‘How’s your wife, and what does she do?’ And then you feel like it’s their assumption, and you just feel like you’re different. I guess it was just an honest mistake, and I guess they didn’t mean anything. They didn’t have any ill intention, but I guess they would’ve been more inclusive and more sensitive when it comes to that…It feels like I don’t belong, like I’m different and stand out in some way, and then that who I am is not the norm.* [Daniel, Asian, 26]

Additionally, participants’ perceptions of their providers’ identities influenced their own communication and disclosure. For example, Mark described how his perception of his provider’s sociodemographic background influenced his comfort level:

*I mean, I think a lot of times I don’t feel like – they either don’t understand gay men’s behavior, especially if I’m going for sexual screening. I just always feel like there’s these barriers to talking with them, especially if like it’s an older white guy who’s clearly straight and just doesn’t understand anything that I’m talking about and can feel slightly judged if I see him or somethin’ like that. So, that’s a barrier.* [Mark, White, 25]

### Theme 2: SMM Express a Strong Desire for Patient-Centered Medical Care

Participants expressed a strong desire for PCC, which is more humanizing and adequately accounts for their lived experiences, including preferring active participation in the construction of care. As Stephen explained:

*I want to participate in the process. I wanna participate in making decisions for my own health, too. I don’t want to just be dictated what I have to do. And so, I like my current doctor because he talks to you in a way that makes you feel like he values your opinion and not that you’re just a patient… I’d prefer my doctors to ask me questions about if I have any concerns about the decision that he or she is making for my health rather than just not asking, just telling me to do it… I want it to be a dialogue instead of, just, you’re gonna* *do this and that.* [Stephen, Asian, 26]

Similarly, Emilio disagreed with the idea that providers should be the sole decision-makers:

*Just because you went to school for this, it doesn’t mean that I’m just gonna… put you in the driver seat and you just gonna drive this car for my health. … you’re gonna do what you have to do make sure that I’m fine, but I’m the one who’s gonna take control. And if I have a question, I’m gonna ask a question, and if I don’t understand something, I’m gonna ask. Because sometimes… we see that well, ‘they’re the doctor, they know best.’ So, whatever they tell you, you’re just taking it. You’re just being receptive to it, as opposed to trying to understand it and going back and forth.* [Emilio, Latinx, 25]

Many participants expressed wanting to feel heard and respected, particularly highlighting the need for individualized care in a comfortable environment. Additionally, many highlighted the importance of developing rapport with their provider. Tommy described how his provider established PCC:

*She talked to me more like a friend than a patient. I didn’t feel judged, like, in anything I was telling her. And, like, it was just like she was a very friendly person... I hate people who are, like, really kind of a bit uptight and, like, they're just straight to the point, just, like, it feels robotic to me, like, you’re just doing your job, like, with the other one, you* *feel a comfort level.* [Tommy, Asian, 25]

### Theme 3: SMM Recognize That LGBTQ-Affirming Providers Facilitate PCC

Participants consistently reported wanting providers with knowledge about and experience with caring for SMM, though opinions varied on whether an LGBTQ-identifying provider would improve their healthcare experiences. Those with a preference for a LGBTQ-identifying provider felt it would facilitate trust and lead to better overall communication. Participants who had seen an LGBTQ-identifying provider described these experiences as affirming and overall satisfying. For example, Max’s relationship with his LGBTQ-identifying provider allowed him to ask potentially stigmatizing questions about various sexual behaviors:

*I feel like my primary care doctor, I’ve asked him questions that… like, Google won’t provide you, like, medical advice on and seemed too, like, stupid to just ask friends. One time, I asked, and I was like, ‘Swallowing cum, what are the sex risks from that?’ I mean the same with, like, rimming where it’s like, you don’t Google that because you’re gonna get a whole bunch of things, where it was, like,* *just very easy to talk to him about that, you know?* [Max, White, 26]

Similarly, Henry said that his provider’s identity helped establish a trusting relationship:

*When my friend told me he had a gay doctor who he really liked, that sounded good and I really like him…I don't think I was searching for [a gay doctor] specifically, but now that I have that, I think that's important for me at least just in terms of even just having a friendly conversational relationship with my doctor kind of like an older gay man. We just talk about movies and Bette Davis and stuff like that whenever I see him. Aside from the healthcare specifically, I like having that kind of relationship*. [Henry, White, 25]

Some participants asserted that they would exclusively see LGBTQ-identified providers, such as Chance, whose preference comes from past experiences with straight providers:

*I’d had not great experiences with straight doctors, so it’s like I just wanted to stick with someone who’s gay who knows what I’m going through, all that kind of stuff, because I had a straight provider that I went to in Chinatown like way back... I went in for STD testing, and I don’t if it was a language barrier, because she’s a doctor but she’s also from a different country. And she kept saying, ‘Well, do you have a girlfriend?’ I was like, ‘No.’ It was just a weird experience… actually I already mentioned that ‘No, I’m actually gay,’ but she still insisted on saying ‘girlfriend.’ ... It was uncomfortable, and I literally left in the middle of the check-up.* [Chance, Asian, 26]

While some participants described preferring LGBTQ-identified providers, others felt that providers’ knowledge and experience in LGBTQ health were more important than identity. Asked about the potential benefits of having a doctor who specializes in SMM, Terrence explained:

*Their knowledge. Their knowledge on the community, their knowledge on our habits, and ways– possible ways of thinking that we do have– It’s much more different than when I was– Like for example, I had one of those pediatric doctors, family doctors. She was a great doctor, I love her to this day, but I didn’t gain the knowledge that I did with* [Name]*, which is the gay men’s health doctor.* *Where he educated us, so there were more on like viral loads, or HIV, or other things and STIs or STDs that we should be cautious about, or certain symptoms that we should look out for. That, just looking out and also giving us the knowledge, or being knowledgeable in some things that particularly relate to me. That was really important. Because you don’t get that a lot. If you go to a doctor, they’re just gonna see you for just your chart, they’ll just look at your history and just go from there. But to be on a personal level, to know me, to understand, to also know… sexual activity, the type of role that I play and what to say, and what knowledge to give me in terms of risks and the percentages and whatever.* *It was really insightful.* [Terrence, Black, 26]

When asked what he looks for in a new doctor, Demetrius explained that shared LGBTQ+ identity can be helpful, but is not the only factor he considers:

*Honestly, that doesn't really matter, but I mean if [the provider] was LGBT, I mean, it's a plus because they could relate more. You know? But as long as they're understanding, and you know, they're like non-judgmental or anything like that,* *like that's another thing that makes me feel comfortable, it makes me feel happy to have that doctor as my main one.* [Demetrius, Black, 25]

Similarly, Jason did not express a preference for LGBTQ-identified providers, explaining that he primarily desires a physician who is LGBTQ-affirming:

*[Having an LGBTQ-affirming doctor] makes it more comfortable for me to talk about my sex life. I just feel like it's a little bit more of a connection I have with my doctors or people I’m talking to, because they’re used to working with LGBTQ people. It’s more of an understanding; I feel, like, a vibe I get from them, but I've had doctors I've been comfortable with that are not, that are not primarily, like, LGBTQ doctors…It's, like, it's not only the comfortability level that, like, they provide, but I feel, like, because it's something they are used to, like, they focus on, they have a little bit more knowledge on, a little bit more understanding on. I don’t know; they just make me feel comfortable. I like to go to a doctor I feel comfortable talking to, especially about my sex life.* [Jason, Asian, 25]

Overall, these results demonstrate how young SMM’s identity and sexual behaviors affect how they navigate medicine. By deepening our understanding of how this marginalized group perceives of the healthcare system, these data can be used to inform interventions that work to give SMM access to care informed by their needs.

## DISCUSSION

This study’s findings expound the need for healthcare providers to focus on population-specific knowledge, cultural humility, and PCC approaches to empower SMM’s healthcare engagement.^67,68^ With numerous health inequities and perceptions of preventive care as unnecessary, it is paramount to optimize implementation of effective and sustainable prevention and treatment strategies for young adult SMM.^23,69,70^ By increasing patients’ security and autonomy in care, PCC could mitigate the harmful effects of healthcare-related stigma and discrimination on the health and well-being of SMM.^66,71^

Participants described experiences in which their providers lacked competence in providing LGBTQ-specific care, citing inadequate knowledge and stigmatizing attitudes. These findings align with the extant literature, which has consistently demonstrated that healthcare professionals do not receive adequate training in treating SMM patients.^30^ Even when providers are comfortable or willing to treat LGBTQ patients, they report lacking the necessary training, knowledge, or skills.^34,51,72–76^ When contextualized within the existing literature, our results underscore that improved education and training for providers is a prerequisite to integrating PCC into clinical practice.‬‬‬‬‬‬‬‬‬‬‬‬‬‬‬‬‬‬‬‬‬‬‬‬‬‬‬‬‬‬‬‬‬‬‬‬‬‬‬‬‬‬‬‬

In addition to wanting more educated providers, participants frequently expressed a desire to be active in the co-construction and management of their care. By prioritizing a collaborative relationship between patients and providers, PCC could help mitigate some of the impacts of health inequities on SMM. Specifically, shared decision-making has shown potential in helping to provide individualized and affirming evidence-based care to LGBTQ individuals,^61,77^ such as through increased adherence to HIV antiretroviral therapy and better management of other chronic diseases.^56,78^ As part of their ideal participation in decision-making, many participants expressed a desire to be heard and respected by providers. This aligns with principals of cultural humility in medicine—an approach that goes beyond cultural competence to position patients as experts in their own bodies and experiences—which can improve healthcare experiences and reduce health inequities for LGBTQ populations.^68,79–82^ While increasing providers’ knowledge about the unique needs of SMM is essential, it will not be sufficient without improving their cultural humility, which is another necessity for implementation of PCC.

While there was a clear consensus on the need for knowledge and humility among providers, many participants stated that a shared identity with their provider would further facilitate communication, disclosure, and informed decision-making. Limited research has investigated the role of patient-provider sexual identity concordance, primarily focusing on patients’ preferences rather than outcomes.^23,83^ However, previous research on patient-provider identity concordance, which has heavily focused on race and gender, has consistently found improved patient satisfaction and health outcomes associated with identity concordance.^84–88^ As there is limited knowledge specific to the role of sexual orientation concordance, future research should explore this with attention to the context of PCC.

Providers must be competent in the knowledge and clinical skills required to care for SMM so that patients who identify as SMM can get clear and accurate information about their health. Without such a foundation, PCC will have limited reach, as providers must be able to participate in shared decision-making to provide appropriate education and recommendations. While patients’ lived experience should be recognized as expertise, providers cannot expect SMM to be experts in clinical care. For example, clinicians must obtain an affirming sexual history—which will be more accurate in patient-centered encounters—to understand what testing may need to be done (e.g., three-site testing for gonorrhea and chlamydia), and then should either follow up with the testing or have an informed, affirming referral. SMM are sensitized to providers who miss these steps or avoid their specific health concerns, and as such may avoid care in the future if they do not receive appropriate treatment.

Beyond skills and knowledge, providing SMM-affirming PCC involves clinicians’ attitudes and behaviors towards SMM. As SMM often avoid healthcare settings, which are already perceived as unwelcoming, clinicians must recognize and avoid the use of stigmatizing or heteronormative language when SMM do present to clinical settings.^89^ Further, the use of affirming language regarding patient’s sexuality and sexual behaviors is required. Asking open-ended questions and reflecting patients’ terminology may enable SMM to feel comfortable engaging in shared decision-making about their sexual healthcare. Such cultural humility and PCC may help to counteract the effects of past negative healthcare experiences on delaying and avoiding future care.

### Limitations

There are several limitations to note. First, this study took place in NYC, where participants generally have increased access to LGBTQ-tailored healthcare providers and city/state-funded resources to obtain low-cost or free sexual healthcare in comparison to people in other parts of the USA. Thus, while the findings presented here are indeed concerning, the study’s location may suggest that young sexual minority men in parts of the USA with less robust public health infrastructure likely face increased challenges when engaging in medical care. Second, while this study and the parent study included sexual minority men and transgender people assigned male at birth, we caution against conflating these populations. We included these participants in the analysis to honor their time and experiences, but the number of transgender individuals in the study is inadequate to draw meaningful conclusions about their unique lived experiences. In public health and medical research, it is paramount that transgender voices be considered in their own right and not obscured by those of cisgender SMM. Related, future research should also explore how transgender men experience medical care, as men who were assigned female at birth may also have unique experiences that warrant tailored and individualized care.

### PCC Implementation Considerations

Despite the potential of PCC approaches, there are many institutional and interpersonal barriers to implementation. For instance, despite widespread use of electronic medical records across healthcare settings, sexual orientation and gender identity are often poorly documented or missing, limiting clinicians’ ability to deliver comprehensive and identity-specific care.^90,91^ Additionally, because the LGBTQ population includes a wide array of racial, ethnic, and cultural identities, integrating PCC requires attention to unique needs of multiply-minoritized patients^102^. For example, limited access to LGBTQ-friendly interpreters, harmful norms around masculinity, and family stigma are a few of the barriers to PCC for Latino SMM,^92,93^ whereas previous discriminatory experiences may hinder identity disclosure and shared decision-making for Black SMM^56^—findings which were reflected in our participants’ experiences. As such, integrative approaches which emphasize the role of intersectionality on SMM’s healthcare experiences could help create more sustainable and affirming interventions.^94^

Finally, comprehensive and widespread training in LGBTQ health is necessary for optimal PCC implementation. Along with medical expertise and communication skills, interventions must emphasize creating inclusive clinical environments; assessing sexual identity and behavior appropriately in clinical encounters; and educating clinical and non-clinical staff on LGBTQ-affirming practices.^30,33,95,96^ Acknowledging and actively pursuing LGBTQ health fluency allows both clinicians and LGBTQ patients to feel more confident and comfortable in their interactions.

## CONCLUSION

The present study demonstrates the need to increase awareness and medical training around patient-centered care, especially for patient populations that often experience discrimination in healthcare settings. Approaches to caring for systematically excluded or marginalized communities must be multidimensional and consider the daily lives of patients as well as the environments in which they live and make decisions about their health.^97^ Clinical and non-clinical interventions that work to enhance and respect SMM’s agency and dignity will help to ameliorate the effects of homophobia and discrimination facing young sexual minority men.^103^
